# Forcible Dilatation of the Sphincter Ani in the Treatment of Hemorrhoids

**Published:** 1877-04

**Authors:** 


Forcible Dilatation of the Sphincter Ani in the Treatment of Hemorrhoids.—Dr. Christofari in his work on this subject comes to the following conclusions:—Contraction of the sphincter ani plays an important role in the production of hemorrhoids. This contraction is much more frequent in hemorrhoidal affection than most writers on the subject think. It produces the constipation and the acute pain which these patients suffer before and after defecation. It exists, too, without pain, and for this should be none the less considered as a cause productive of hemorrhoids. In removing the constriction the physician relieves the painful symptoms which it occasions and at the same time may treat the diseased parts. The best method to employ is that of forcible dilatations. This proceeding, so simple and so harmless, has succeeded in a very great number of cases.—*Gaz. des Hopitaux.*

